# Supplementary material for: Thiosulfinate-Enriched Allium sativum Extract Exhibits Differential Effects between Healthy and Sepsis Patients: The Implication of HIF-1α
Source: Int J Mol Sci. 2023 Mar 25;24(7):6234. doi: 10.3390/ijms24076234 (PMC10094690; doi:10.3390/ijms24076234)
Supplement: Supplementary file 1 [file ijms-24-06234-s001.zip › ijms-2266052-supplementary.pdf]

SUPPLEMENTAL INFORMATION

Thiosulfinate-Enriched *Allium sativum* extract exhibits differential effects between healthy and sepsis patients: the implication of HIF-1α

José Avendaño-Ortiz, Francisco Javier Redondo-Calvo, Roberto Lozano-Rodríguez, Verónica Terrón-Arcos, Marta Bergón-Gutiérrez, Concepción Rodríguez-Jiménez, Juan Francisco Rodríguez, Rosa del Campo, Luis Antonio Gómez, Natalia Bejarano-Ramírez, José Manuel Pérez-Ortiz, Eduardo López-Collazo.

Supplementary Figure S1

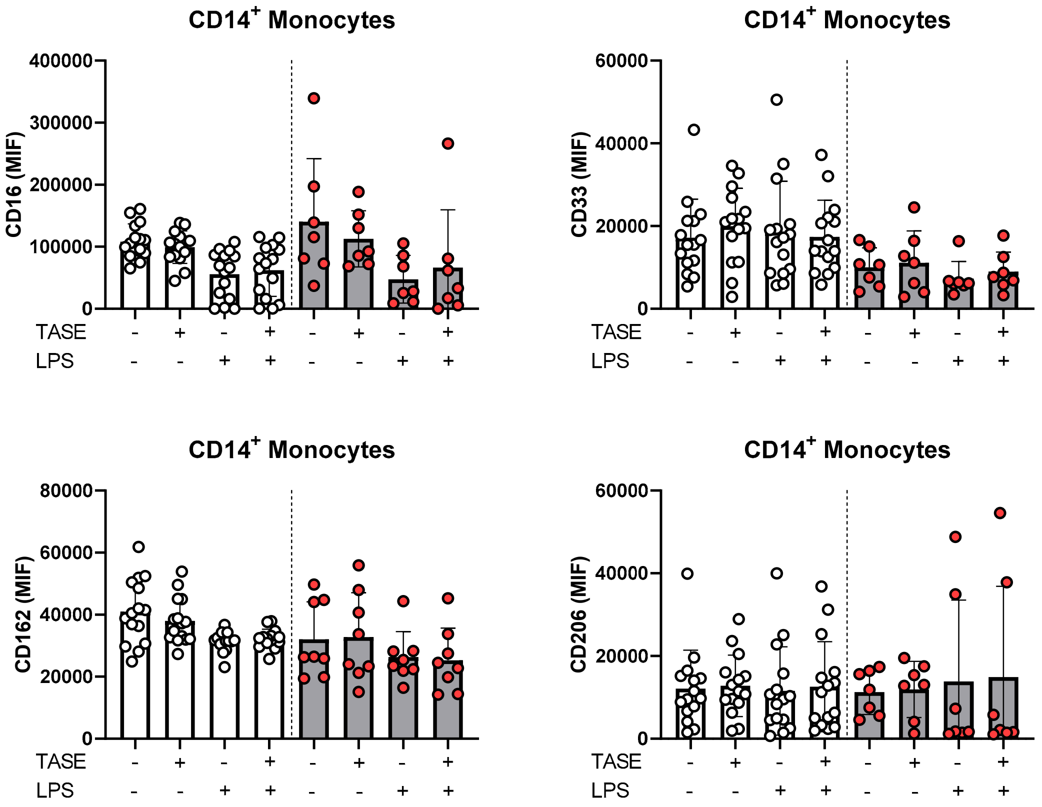

Supplementary Figure S1. Immune markers expression in monocytes from both HV and sepsis patients treated with garlic extract and LPS. Monocytes from HV (n=15) and sepsis (n=7) were stimulated with 10 ng/mL for 16 h in combination or not with 3 µg/mL of thiosulfinate-enriched Allium sativum extract (TASE) and labelled with cytometry antibodies. Mean intensities of fluorescence of CD16, CD33, CD162 and CD206 on gated CD14+ monocytes determined by flow cytometry are shown. Dots show individual values and bars express mean±SD.

Supplementary Figure S2

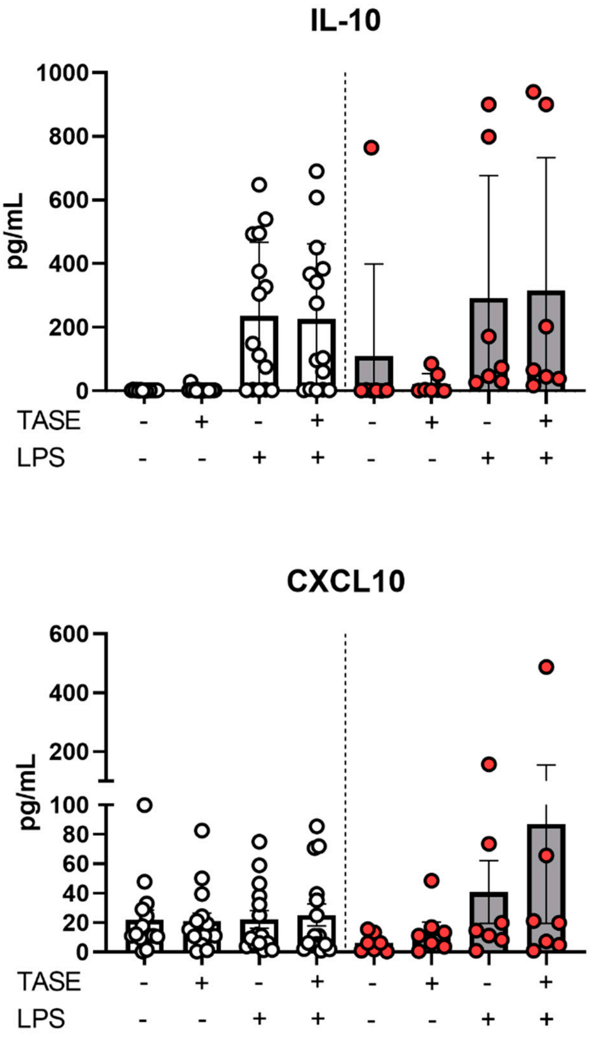

Supplementary Figure S2. Cytokine production in monocytes from both HV and sepsis patients treated with garlic extract and LPS. Monocytes from HV (n=15) and sepsis (n=7) were stimulated with 10 ng/mL of LPS for 16 h in combination or not with 3 µg/mL of thiosulfinate-enriched Allium sativum extract (TASE). Inflammatory cytokine levels of IL-10 and CXCL10 in cell culture supernatant were measured. Concentrations are shown. *, p<0.05 in paired Wilcoxon t test. Dots show individual values and bars express mean±SD.

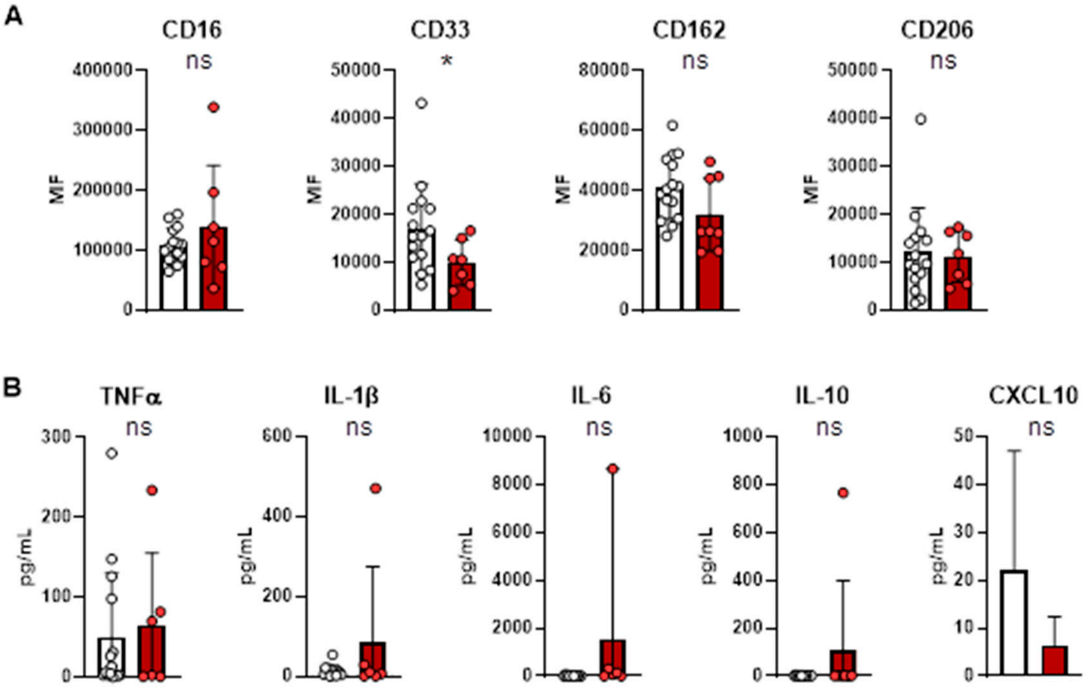

Supplementary Figure S3. Membrane marker expression and cytokine levels in supernatants of monocytes from both HV and sepsis patients in basal control condition. Monocytes from HV (n=15) and sepsis (n=7) were culture with complete RMPI and leave untreated for 16 h. A. Mean intensities of fluorescence of CD16, CD33, CD162 and CD206 on gated CD14+ monocytes determined by flow cytometry are shown. B. Levels of TNFα, IL-1β, IL-6, IL-10 and CXCL10 in cell culture supernatant are shown. *, *$p<0.05$* and ns, non-significant in unpaired t test. Dots show individual values and bars express mean±SD.
